# Clinical and Genetic Heterogeneity of *CARD14* Mutations in Psoriatic Skin Disease

**DOI:** 10.3389/fimmu.2018.02239

**Published:** 2018-10-16

**Authors:** Laura Israel, Mark Mellett

**Affiliations:** ^1^Novartis Institutes for BioMedical Research, Novartis Campus, Basel, Switzerland; ^2^Department of Dermatology, University Hospital Zürich, Zürich, Switzerland

**Keywords:** CARD14 (CARMA2), gain-of-function (GoF) mutation, keratinocytes, skin inflammation, psoriasis, pityriasis rubra pilaris

## Abstract

The CARD: BCL10: MALT1 (CBM) complex is an essential signaling node for maintaining both innate and adaptive immune responses. CBM complex components have gained considerable interest due to the dramatic effects of associated mutations in causing severe lymphomas, immunodeficiencies, carcinomas and inflammatory disease. While MALT1 and BCL10 are ubiquitous proteins, the CARD-containing proteins differ in their tissue expression. CARD14 is primarily expressed in keratinocytes. The CARD14-BCL10-MALT1 complex is activated by upstream pathogen-associated molecular pattern-recognition *in vitro*, highlighting a potentially crucial role in innate immune defense at the epidermal barrier. Recent findings have demonstrated how CARD14 orchestrates activation of the NF-κB and MAPK signaling pathways *via* recruitment of BCL10 and MALT1, leading to the upregulation of pro-inflammatory genes encoding IL-36γ, IL-8, Ccl20 and anti-microbial peptides. Following the identification of CARD14 gain-of function mutations as responsible for the psoriasis susceptibility locus *PSORS2*, the past years have witnessed a large volume of case reports and association studies describing CARD14 variants as causal or predisposing to a wide range of inflammatory skin disorders. Recent publications of mouse models also helped to better understand the physiological contribution of CARD14 to psoriasis pathogenesis. In this review, we summarize the clinical, genetic and functional aspects of human and murine CARD14 mutations and their contribution to psoriatic disease pathogenesis.

## Introduction

Caspase Recruitment Domain-containing protein 14 (CARD14) (also called CARMA2 or BIMP2) is the second member of the CARMA family that consists of CARD11 (CARMA1), CARD10 (CARMA3) and related molecule CARD9 (1). Like its family members, CARD14 recruits interacting partners BCL10 and MALT1 (2–4) to form the so-called CBM complex and thus initiate NF-κB and MAPK signaling pathways. Therefore, the CARMA proteins trigger a shared downstream pathway and are, with the exception of CARD9, highly similar in sequence and structure (1, 3). BCL10 and MALT1 are ubiquitously expressed proteins whereas the CARD molecule gives cell specificity to the complex, with CARD11 localizing to lymphoid cells and tissue, CARD9 being restricted to myeloid cells, CARD14 in skin and mucosal tissues, whereas CARD10 displays a broader expression in epithelial and endothelial tissue (1, 5, 6). Mutations associated with the CARMA family proteins therefore result in very different human maladies (7, 8).

CARD14 was originally identified in placental tissue (3) but microarray data later revealed that the molecule is highly expressed in adipose tissue, esophagus and mucosal tissues (1). In 2012, first reports from the Bowcock group described autosomal dominant CARD14 gain-of-function (GoF) mutations in a young patient presenting with a severe form of pustular psoriasis and in two multiplex families with plaque psoriasis (9, 10). Additionally, CARD14 was discovered to be highly expressed in keratinocytes and was identified as the causative gene at the *Psoriasis Susceptibility* (*PSORS*) *2* locus, which had previously been identified as one of the principle risk loci for psoriasis (11). Furthermore, a report from the Sprecher group broadened the occurrence of CARD14 mutations to pityriasis rubra pilaris (PRP), a rarer form of psoriatic skin disease (12). These studies were the first to reveal a pathogenic role for CARD14 and also highlighted the physiological variation and scope of the CARMA protein family's influence to human health and disease.

Since these inaugural discoveries, further studies have revealed CARD14 variants associated with various entities in the psoriasis disease spectrum. Of particular note is the considerable clinical heterogeneity associated with CARD14 mutations in terms of phenotype and severity, even within a single disease entity. In this review, we discuss the subtypes of the psoriatic disease spectrum, which have been described to be associated with CARD14 mutations and seek to differentiate between causal and predisposing variants.

## Inflammatory skin disorders associated with CARD14 variants

### Psoriasis

#### Clinical features

Psoriasis is a common chronic inflammatory skin disease with a complex genetic background affecting approximately 2% of the global population (13) though this varies depending on ethnicity, demographics and latitude (14). It is now well established that psoriasis is a multigenic disease that arises in genetically susceptible individuals in response to an environmental trigger, such as skin trauma or infection (15). Immunopathogenesis is orchestrated by a complex interplay between keratinocytes, skin resident immune cells and infiltrating leukocytes, including neutrophils, macrophages, conventional and plasmacytoid dendritic cells. Infiltrating type 1 and type 17 helper T cells (Th1/Th17) cells maintain the chronic inflammation associated with established disease (16). The term “psoriasis” envelopes various forms, which are typically classified by morphology, distribution and anatomical localisation (17). Major distinctions include plaque and pustular forms, which contrast in appearance and with the immune cell infiltrate involved.

Plaque psoriasis [or psoriasis vulgaris (PsV)] is the most common form of psoriasis, accounting for approximately 80–90% of psoriatic cases (18), it is characterized by raised demarcated erythematous circular plaques on the skin of sufferers, due to hyperproliferation of keratinocytes (15), leading to thickening of the skin (acanthosis). Plaque psoriasis is also associated with dilation and increased number of blood vessels, facilitating the immune cell infiltration and maintaining chronicity.

Comorbidities including cardiovascular disease, Crohn's disease, obesity and metabolic syndrome have been linked with PsV (19–21). Additionally, 25% of cases are associated with psoriatic arthritis, though this can also precede the skin condition in some patients (22). These comorbidities emphasize the systemic nature of psoriasis with effects spreading beyond the skin. The link with comorbidities is not well understood, though it has been proposed that elevated levels of circulating pro-inflammatory cytokines found in the serum of patients, in particular IL-17A, foster these systemic abnormalities in secondary organs (23–25).

Pustular psoriasis is characterized by skin eruptions of white pustules and surrounding red erythematous skin and can take both localized and generalized forms. Infiltrating neutrophils and monocytes to skin tissue is a hallmark of the disease entity. Skin flares in generalized pustular psoriasis (GPP) can be accompanied by high fever, fatigue and muscle and joint pain and secondary effects include acute respiratory disease, uveitis, osteoarthritis and cholangitis of the bile ducts, which is mediated by neutrophilic activation (26). Patients with GPP carrying loss-of-function (LoF) mutations in *IL36RN* (encoding IL-36 receptor antagonist) or heterozygous mutations in *AP1S3* (resulting in increased IL-36α expression) were described, suggesting a pivotal role of IL-36 cytokine activity in driving pustular psoriasis (27–31).

#### Genetics

**Figure 1:**
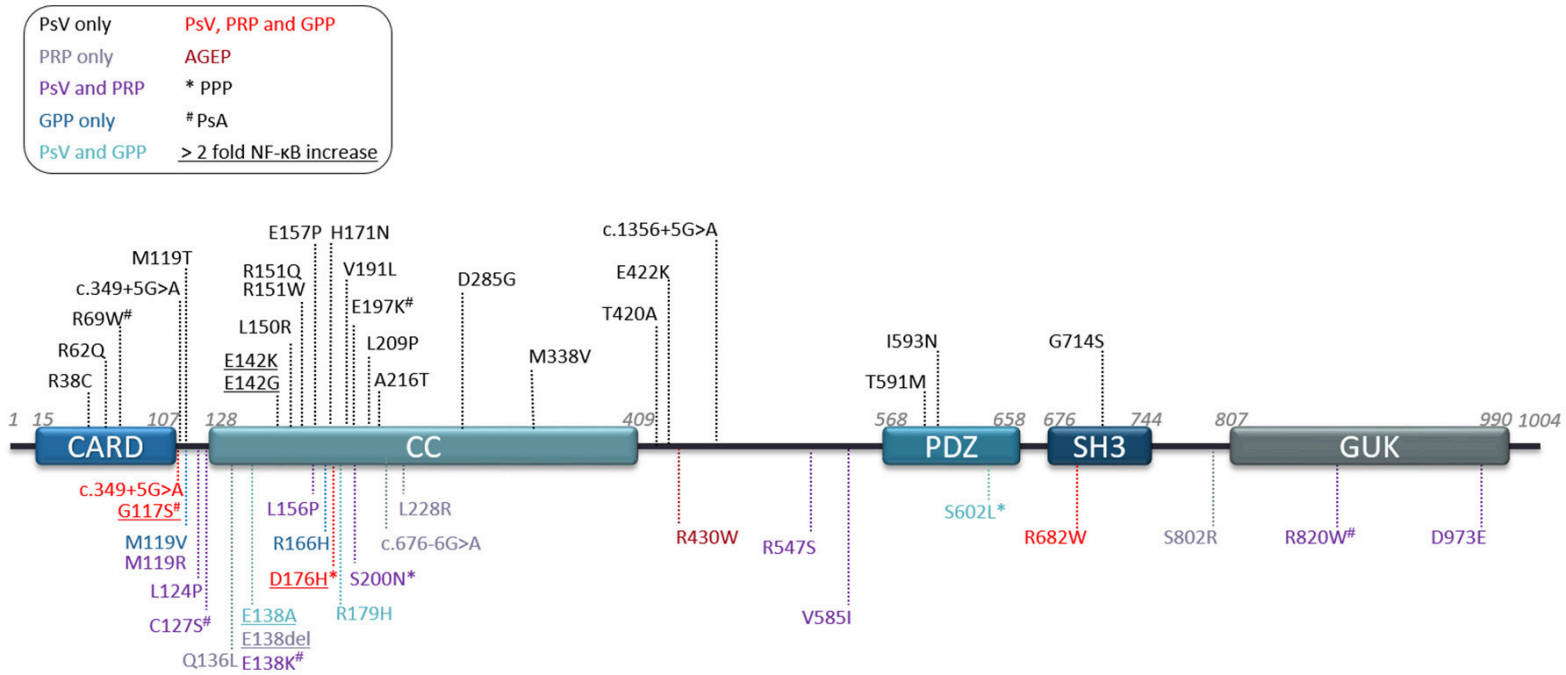
Human CARD14 protein domains and reported variants. Schematic depicting protein domains of the human CARD14 protein and location of all variants reported to date. Legend key shows disease association by color and reports of associated palmoplantar pustular psoriasis (*PPP) or psoriatic arthritis (^#^PsA). Underlined variants are mutants studied in terms of NF-κB activation in overexpression studies and showing at least 2 fold more induction than CARD14-WT. AGEP, Acute generalized exanthematous pustulosis; GPP, generalized pustular psoriasis; PRP, pityriasis rubra pilaris; PsV, psoriasis vulgaris.

Genome-wide linkage analysis studies identified nine “Psoriasis Susceptibility” regions or loci (*PSORS1-9*) (11), however only *PSORS1, -2* and *-4* findings were replicated in independent studies (32). In 1994, the psoriasis susceptibility locus (*PSORS2* ([MIM 602723]) was mapped at the distal end of human chromosome 17q in a large Caucasian kindred with several affected generation members presenting with plaque type psoriasis (33) and this was confirmed by other studies, in particular in a five-generation Taiwanese family with psoriasis (34, 35). Extensive analysis of these two families by Jordan et al., led to the identification of *CARD14* as being the responsible gene for the underlying association of the *PSORS2* locus with psoriasis (9). In the family of European descent, a heterozygous missense mutation (p.G117S) was identified, whereas in the Taiwanese kindred an intronic mutation (c.349 + 5G>A) was found. Both these variants created a cryptic splice site resulting in a 22 amino acid insertion between exons 3 and 4. In addition, Jordan et al. identified a *de novo* mutation (p.E138A) in a young patient, originating from Haiti, presenting with early-onset GPP (Table 1 and Figure 1). By screening seven psoriasis cohorts with varying ancestries (over 6,000 cases and 4,000 controls), Jordan and colleagues also identified 15 additional rare and common CARD14 variants that were enriched in cases over controls (10) (Tables 1, 2).

**Table 1:** CARD14 Gain-of-function mutants associated with psoriatic skin disease.

**Protein**	**mRNA**	**SNP**	**MAF in ExAC**	**Disease**	**Penetrance**	**Inheritance**	**Zygosity**	**Co factor**	**References**
G117S	c.349G>A	rs281875215	not found	PsV	complete	AD	Het	no HLA-Cw(06:02)	(9)
				PsV			Het		(10)
				PsV	Incomplete (1 healthy carrier)	AD	Het		(36)
				PsV					(37)
				PsV and GPP	Incomplete (1 healthy carrier)	AD	cpd Het	other CARD14 variants HLA-Cw(06:02)	(40)
				GPP			Het	IL36RN Het mutation (S113L)	(76)
				PRP type V	Incomplete (1 healthy carrier)	AD	Het		(56)
				CAPE	complete	AD	Hom		(39)
splice	c.349 + 1G>A	rs886041402	not found	familial PRP	incomplete	AD	Het		(12)
				GPP familial	Incomplete (2 healthy carriers)	AD	Het		(45)
splice	c.349+5G>A	rs587777763	not found	PsV	complete	AD	Het	no HLA-Cw(06:02)	(9)
				CAPE		AD	Het		(39)
E138K	c.412G>A		not found	erythro PRP		*de novo*	Het		(58)
				PRP type V sporadic		*de novo*	Het		(59)
				CAPE		*de novo*	Het		(39)
E138A	c.413A>C	rs281875214	not found	GPP		*de novo*	Het	no IL36RN/no HLA-Cw(06:02)	(9)
				PsV			Het		(10)
E138del	c.412_414delGAG		not found	familial PRP	incomplete	AD	Het		(12)
E142K	c.424G>A	rs281875212	not found	PsV			Het		(10)
E142G	c.425A>G	rs281875213	not found	PsV			Het		(10)
L156P	c.467T>C	rs387907240	not found	familial PRP	complete	AD	Het		(12)
				CAPE	complete	AD	Het		(39)
H171N	c.511C>A	rs281875216	not found	PsV			Het		(10)
D176H	c.526G>C	rs144475004	0.00495	PsV			Het		(10)
				GPP with PsV			Het	no IL36RN/no HLA-Cw(06:02)	(44)
				PsV	found in controls				(49)
				GPP			Het		(38)
				PPP			Het	no HLA-Cw(06:02)	(41)
				PsV			Het		(51)
				PPP			Het		(76)
				PRP type I and IV			Het		(56)

*CARD14 mutations that were described as GoF based on in vitro functional studies*.

*AD, autosomal dominant; CAPE, CARD14-associated papulosquamous; CC, coiled-coil; GPP, generalized pustular psoriasis; Het, heterozygous; Hom, homozygous; PPP. Palmoplantar pustular psoriasis; PRP, pityriasis rubra pilaris; PsV, psoriasis vulgaris; Cpd, compound*.

**Table 2:** CARD14 variants associated with psoriatic skin disease.

									**Cofactors**			
**Prot**.	**mRNA**	**SNP**	**MAF in ExAC**	**Disease**	**Origin**	**Cohort size case/ctr**	**MAF CASE**	**MAF CTR**	**Other CARD14**	**HLA-Cw0602**	**Treatment/Response**	**References**
R38C	c.112C>T	rs281875217	not found	PsV	Caucasian/Asian	6000/4000	0.00019	0				(10)
R62Q	c.185G>A	rs115582620	0.002606	PsV	Caucasian/Asian	6000/4000	0.0014	0.00084				(10)
				PsV	Tunisian	282/192	0.0053	0				(37)
R69W	c.205C>T	rs375624435	0.0001945	PsV	Tunisian	282/192	0.008865	0				(37)
M119 R	c.356T>G		not found	CAPE		15 kindreds	1 patient				etanercept/partial	(39)
				familial PRP		case report (2 Pts)					ustekinumab/comple te	(61)
M119T	c.356T>C		not found	CAPE		15 kindreds	2 patients					(39)
M119V	c.355A>G		not found	GPP	Han Chinese	174 PsV,62 GPP/365 C	0.002 (GPP)	0				(49)
				CAPE		15 kindreds	1 patient				ustekinumab/complete	(39)
L124P	c.371T>C		not found	familial PRP		case report (1 kindred)					ustekinumab/complete	(57)
				familial PRP, Psv		case report (1 kindred)			yes	yes		(42)
C127S	c.380G>C		not found	CAPE		15 kindreds	1 kindred				MTX and etanercept/partial	(39)
				PRP type V.		22/100	0.0227	0				(56)
Q136L	c.407A>T		not found	PRP type V		22/100	0.0227	0				(56)
L150R	c.449T>G	rs146214639	0.001859	PsV	Caucasian/Asian	6000/4000	0.0025	0.0016				(10)
				PsV	Tunisian	282/192	0.001773	0				(37)
R151Q	c.452G>A	rs200731780	0.0003399	PsV	Tunisian	282/192	0.001773	0				(37)
R151W	c.451C>T	rs777305616	4.217E-05	PsV	European							(37)
Q157P	c.470A>C		not found	CAPE		15 kindreds	1 patient				MTX/partial	(39)
R166H	c.497G>A		not found	GPP	Han Chinese	174 PsV,62 GPP/365 C	0.002 (GPP)	0				(49)
R179H	c.536G>A	rs199517469	0.0002708	PsV	Caucasian/Asian	6000/4000	0.00025	0.00027				(10)
				GPP		51 patients						(76)
V191L	c.571G>T	rs281875218	not found	PsV	Caucasian/Asian	6000/4000	0.00014	0.00027				(10)
E197K	c.589G>A	rs200790561	0.000776	PsV	Tunisian	282/192	0.01241	0.002604				(37)
S200N	c.599G>A	rs114688446	0.01096	PRP		48 patients	0.03125					(60)
				PsV	Caucasian/Asian	6000/4000	0.011	0.0084				(10)
				PsV	Tunisian	282/192	0.003546	0				(37)
				PPP		251/1054	0.01195	0.00332		no		(41)
L209P	c.626T>C		not found	PsV	European							(37)
A216T	c.646G>A	rs574982768	5.798E-05	PsV	Han Chinese	174 PsV,62 GPP/ 365 C	0.002 (PsV)	0				(49)
				PsV	European							(37)
				PsV	Han Chinese	131/207	0.0076	0				(51)
splice	c.676-6G>A	rs28674001	0.3481	sporadic PRP	Hungarian	case report			yes			(81)
L228R	c.683T>G	rs142246283	9.359E-05	PRP		48 patients	0.01042					(60)
D285G	c.854A>G	rs281875219	not found	PsV	Caucasian/Asian	6000/4000	0.00019	0				(10)
M338V	c.1012A>G	rs200132496	9.074E-05	PsV	Tunisian	282/192	0.003546	0				(37)
T420A	c.1258A>G	rs762364495	not found	PsV	Tunisian	282/192	0.001773	0				(37)
E422K	c.1264G>A	rs61751629	not found	PsV, anti TNF response		116 patients (79 resp,37 non resp)	0.04 (resp)	0 (non resp)			anti TNF/positive	(48)
R430W	c.1288C>T		6.673E-05	AGEP	Spanish	case report (1 Pt)						(90)
splice	c.1356+5G>A	rs376524884	0.0001012	PsV	Tunisian	282/192	0.001773	0				(37)
R547S	c.1641G>C	rs2066964	0.3772	PsV	Yemenite Ashkenazi	case report (1 kindred)			yes	yes		(40)
				PsV	Han Chinese	174 PsV,62 GPP/ 365 C	0.441	0.46				(49)
				sporadic PRP	Taiwanese	case reports (8 patients)			yes			(52)
				familial PRP, Psv		case report (1 kindred)			yes	yes		(42)
				sporadic PRP	Hungarian	case report			yes			(81)
V585I	c.1753G>A	rs34367357	0.08418	PsV	Yemenite Ashkenazi	case report (1 kindred)			yes	yes		(40)
				PsV	Han Chinese	174 PsV,62 GPP/365 C	0.074	0.056				(49)
				familial PRP, Psv		case report (1 kindred)			yes	yes		(42)
												
T591M	c.1772C>T	rs200102454	0.0003674	PsV	Han Chinese	174 PsV,62 GPP/ 365 C	0.002 (PV)	0				(49)
I593N	c.1778T>A		not found	PsV	Caucasian/Asian	6000/4000	0.00024	0.00048				(10)
S602L	c.1805C>T	rs201285077	0.0002346	GPP	European							(37)
R682W	c.2044C>T	rs117918077	0.01103	PsV	Caucasian/Asian	6000/4000	0.013	0.012				(10)
				GPP	Han Chinese	174 PsV,62 GPP/ 365 C	0.002 (GPP)	0				(49)
				PsV, anti TNF response		116 patients (79 resp,37 non resp)	0.01 (resp)	0 (non resp)			anti TNF/positive	(48)
				PRP		48 patients	0.01042					(60)
				sporadic PRP	Hungarian	case report			yes			(81)
G714S	c.2140G>A	rs151150961	0.000423	PsV	Caucasian/Asian	6,000/4,000	0.0021	0.0014				(10)
S802R	c.2406C>A		not found	PRP		48 patients	0.01042					(60)
R820W	c.2458C>T	rs11652075	0.4244	PsV and PRP	Caucasian/Asian	6,000/4,000	common variant			yes		(10)
				PsV and PA	Spanish	400/420	0.4375	0.5214				(47)
				PsV	Han Chinese	174 PsV,62 GPP/365 C	0.443	0.482				(49)
				PsA	Japanese	case report (1 Pt)						(43)
				PsV, anti TNF response		116 patients (79 resp,37 non resp)	0.55 (in resp)	0.38 (non resp)			anti TNF/positive	(48)
				PsV	Caucasian/Asian	32,807/45,458						(50)
				PsV	Yemenite Ashkenazi	case report (1 kindred)			yes	yes		(40)
				sporadic PRP	Taiwanese	case reports (8 Pt)			yes			(52)
				familial PRP, Psv		case report (1 kindred)			yes	yes		(42)
				sporadic PRP	Hungarian	case report			yes			(81)
D973E	c.2919C>G	rs144285237	0.003435	PsV and PRP	Caucasian/Asian	6,000/4,000	0.0024	0.0015				(10)

*Reported variants described in CARD14 excluding pathogenic mutants (Table 1). CARD14 variants here have not been studied functionally or did not appear GoF in vitro*.

*AGEP, Acute generalized exanthematous pustulosis eruption; CAPE, CARD14-associated papulosquamous; GPP, generalized pustular psoriasis; PRP, pityriasis rubra pilaris; PsV, psoriasis vulgaris; Pts, patients; Resp, responder; Non resp, non-responder; MTX, methotrexate*.

Following these initial studies, CARD14 variants were reported by several groups as causal or predisposing factors for psoriasis with or without psoriatic arthritis or palmoplantar pustular psoriasis (PPP) (36–51). Together, these studies indicate a diverse range of disease symptoms associated with CARD14 mutations. However, dissecting whether CARD14 variants function as causal mutations or predisposing factors has become difficult to interpret.

The majority of CARD14 variants were found in the heterozygous state. However, a few patients were described with compound heterozygous (40, 42, 52) or homozygous variants (39, 43) nevertheless, these were not associated with a more severe phenotype. CARD14 variants are reported in exons 2, 3, 4, 6, 7, 9, 13, 15, 18, and 21, but exon 3 and 4 (encoding partly for the CARD and Coiled-coil (CC) domains) represent a clear hotspot with 63% of published variants (Figure 1). Some of these mutations are synonymous with CARD11 mutants, which have been described to affect the autoinhibitory conformation that impedes recruitment of BCL10 (53). These mutations result in spontaneous or sustained activation of downstream NF-κB and MAPK pathways.

#### Treatments

Psoriasis runs a chronic course and current treatments alleviate symptoms rather than treat causative drivers at the root of disease pathogenesis. Traditionally treatments include methotrexate, corticosteroids, UV phototherapy for mild cases, Acitretin a second-generation retinoid, Vitamin D analogs and also coal tar (54). Recently, biologic therapy has expanded beyond TNFα inhibitors to IL-12/IL-23p40-, IL-17A-, IL-17RA-, and IL-23p19-targeting antibodies, which have demonstrated increased efficacy in clinical trials compared to TNF blockade [reviewed elsewhere (55)]. It remains of interest to determine whether targeting the IL-23/Th17 axis will also be beneficial in alleviating associated comorbidities.

To assess whether CARD14 variants could be associated with better responses in psoriasis patients to anti-TNF therapy, Coto-Segura and colleagues tested the presence of such variants in 116 patients who underwent anti-TNF treatment (48). These patients were all previous non-responders to classical psoriasis treatments (phototherapy, methotrexate, acitretin or cyclosporine). A higher frequency of CARD14 variants, in particular the common p.R820W polymorphism, were found among responders to TNFα-targeted blockade, suggesting that CARD14-associated psoriasis could benefit from anti-TNF treatment. Takeichi et al. also reported beneficial treatment with anti-TNF therapy in CARD14-associated familial GPP (56). Additionally, Craiglow et al. described a beneficial outcome upon treatment with ustekinumab (anti-IL-12/IL-23p40) in CARD14-associated psoriasis in individual patients (39).

### Pityriasis rubra pilaris (PRP)

#### Clinical features

CARD14 variants were also found to be associated with another inflammatory skin disorder related to psoriasis, called pityriasis rubra pilaris (PRP) (12, 52, 56–61). PRP is an extremely rare inflammatory skin disease with an estimated prevalence of 1/400,000 individuals (62). It is characterized by scaly salmon-colored (“rubra”) plaques on the skin of affected individuals and though a separate entity to psoriasis the two diseases share overlapping clinical features, which makes ambiguous cases of PRP challenging to diagnose. Typically, clinical and histological features enable correct identification of PRP; these include the presence of “sparing-islands” of uninvolved skin, and the presence of distinctive alternating ortho- and parakeratosis (63).

PRP has a bimodal age-of-onset distribution and is subclassified into 6 types. The most common type (type I, approximately 50% of cases) initially affects both men and women in their late fifties and sixties, while other forms may have a juvenile or neonatal onset. The latter cases are particularly associated with familial PRP (type V) and present with a deleterious form that runs a chronic course and is refractory to conventional psoriasis treatments (12, 62, 64). Familial cases have been associated with *CARD14* GoF mutations and *CARD14* mutant variants have also been described in other sporadic forms (52, 56).

Besides *CARD14* mutations, other causal factors of PRP remain elusive. Interestingly, cases have been reported to be preceded by bacterial or viral infections (65–67) and indeed HIV-associated PRP has been classified as a distinct type of PRP (type VI) (68, 69).

Like psoriasis, PRP also presents with associated comorbidities including hypothyroidism, dyslipidaemia and the most common form, classical adult onset (type I) has been associated with underlying malignancies (62, 69, 70).

#### Genetics

Several groups reported CARD14 mutations in patients with both familial and sporadic PRP (12, 42, 52, 56–59, 61) (Tables 1, 2). Indeed, PRP type V is considered a PRP subtype caused by CARD14 mutations (56). Interestingly, Craiglow et al. recently described 15 patients with CARD14 mutations that present with both psoriasis and PRP symptoms, and grouped these skin inflammatory phenotypes under the appellation *CARD14-associated papulosquamous eruption* (CAPE) (39). CAPE is characterized by distinctive facial plaques on the cheeks, chin and upper lip with absence below the lower lip. Erythema of the ears, trunk and extremity involvement and palmoplantar keratoderma are also present.

As with psoriasis, most CARD14 variants associated with PRP are heterozygous, with very few compound heterozygous patients having being described (42, 52). CARD14 variants associated with PRP, like psoriasis, are spread throughout the protein domains (Figure 1) but also concentrate in exons 3 and 4. To date, no correlation has been made between variant localisation and disease severity or age of onset. However, mutations in this area affect the ability of the molecule to maintain its autoinhibitory state (2, 71–73).

#### Treatments

First-line treatments of PRP include oral retinoids and methotrexate, followed by topical corticosteroids or emollients. Difficult cases prove refractory to conventional psoriasis treatments and UV-light therapy can trigger or aggravate symptoms (70). The use of biologic therapy targeting TNFα (infliximab) or IL-12/IL-23p40 (ustekinumab) has proven effective in individual cases (61, 74, 75). Other groups also described beneficial effect upon treatment with ustekinumab in CARD14-associated familial PRP (57, 61).

## Genetic heterogeneity associated with CARD14 variants

In addition to the association of CARD14 variants with different disease entities, it is also noteworthy that several CARD14 variants have been associated with more than one type of skin disease. For example, p.D176H (Table 1), which was initially described in patients with PsV only (10, 51) or PsV with PPP (41, 76), was subsequently also described as associated with GPP but not with PsV in Asian populations (38, 44) and Takeichi et al. also found the p.D176H variant in patients with PRP type V (56). This reflects the broad heterogeneity found in patients with CARD14 mutations. It might also suggest that these particular variants are predisposing factors and require cofactors or environmental triggers that then determine progression of different disease entities. This is supported by the fact that most of CARD14 variants display incomplete penetrance, where healthy carriers may carry protective factors or lack susceptibility genetic cofactors or exposure to environmental stimuli.

*PSORS1* is the locus shown to confer the greatest risk for psoriasis, accounting for 35–50% of heritability (77) and in 2000, HLA-Cw^*^0602 was described as the psoriasis risk allele mapping to this locus (78, 79). Some studies revealed increased evidence for association of CARD14 p.R820W with psoriasis when the study was conditioned on HLA-Cw^*^0602 (10) and one kindred with familial psoriasis associated with CARD14 mutations were shown to be also positive for the HLA-Cw^*^0602 allele (40). On the other hand, patients harboring CARD14 variants were found negative for this allele in other studies (9, 44) or no association was found between CARD14 variants and the *HLA* locus (44). Unfortunately, this locus was not systematically assessed in patients with CARD14-associated psoriasis and further investigation are required to better understand if these loci might act as cofactors in triggering pathology.

In other studies, genetic association between psoriasis and CARD14 is also not clear. Berki et al. analyzed a cohort of 416 PsV patients and were unable to identify any CARD14 variant (38). Suguira and colleagues described a Down syndrome patient with PsA homozygous for the p.R820W CARD14 variant. However, both parents were also homozygous for this variant and at date of publication, had not developed any psoriatic phenotype, supporting the fact that p.R820W is a common variant found in healthy individuals (MAF = 0.4244 in ExAc), which might only predispose to psoriatic skin diseases (43). Finally, Eskin-Schwartz et al., described an interesting kindred with CARD14-related psoriasis with extreme clinical variability (from mild plaque-type to GPP) (40). Affected members were heterozygous for the p.G117S pathogenic CARD14 allele, but most severely affected members were also carrying three additional CARD14 variants (p.R547S, p.V585I, and p.R820W) as well as the HLA-Cw^*^0602 allele. However, one family member is also a healthy carrier of the p.G117S, suggesting incomplete penetrance, also supported by Ammar et al. reporting one healthy carrier for p.G117S (36). This healthy carrier from the Eskin-Schwartz report is negative for the three additional CARD14 variants and for the 06:02 allele at the HLA-C locus, suggesting that genetic cofactors as well as environmental triggers might be required for CARD14-induced psoriasis.

Altogether, these studies indicate that the assessment of linkage to psoriasis susceptibility loci should be made cautiously as a number of factors complicate the analyses, including genetic cofactors, statistical limitations due to studies with narrow sample size, incomplete penetrance, environmental factors, and misdiagnosis.

## Heterogeneous impact of CARD14 mutations at the molecular level

Immunohistochemistry staining and mRNA assessment revealed that CARD14 localizes to the basal cell layer of the epidermis in healthy skin whereas it is found in suprabasal layers in psoriatic tissue (1, 9, 12).

Several CARD14 mutants were studied at the molecular level in terms of their ability to activate NF-κB (Table 3). In reporter gene assays, when compared to WT-CARD14, NF-κB luciferase activity was found strongly increased upon over expression of p.E138A (7–9 fold), p.E138del (2–3 fold), p.E142K/G (4–5 fold), p.G117S (3–4 fold), and p.D176H (2–3 fold); and moderately increased for p.R179H, p.E197K, p.L150R, and p.L228R (1.4–1.7 fold) (Table 3). However, NF-κB activity was found unaffected upon overexpression of p.R68Q, p.V191L, p.D285G, p.M338V, p.I593N, p.S602L, p.R682W, and p.G714S and reduced for variants p.R38C, p.R69W, p.R151Q, p.S200N, p.L209P, p.H171N, p.T420A (0.1–0.7 fold) (9, 10, 37, 60). Other variants were not assessed in terms of NF- κB luciferase activity.

**Table 3:** Functional characterization of CARD14 variants.

**Protein Change**	**CARD14 domain**	**NF-κB reporter**	**Pathogenic signature upon O/E**	**Other functional testing**
R38C	CARD	0.11	no	
R62Q	CARD	1.06	no	
R69W	CARD	0.144	nd	
G117S	Between CARD-CC	3.71	yes	- over expressed in EC in psoriatic skin - no spontaneous oligomerization - increased interaction with MALT1 and BCL10, increased ERK/p38 activation
M119R	Between CARD-CC	nd	nd	
M119T	Between	nd	nd	
	CARD-CC			
M119V	Between	nd	nd	
	CARD-CC			
L124P	Between CARD-CC	nd	nd	
C127S	Between CARD-CC	nd	nd	
Q136L	CC	nd	nd	
E138K	CC	nd	nd	
E138A	CC	8.95	yes	- increased CARD14 staining and proinfl. gene expression in lesional skin - over expressed in EC in psoriatic skin - spontaneous CARD14 oligomerization - increased interaction with MALT1 and BCL10, increased ERK/p38 activation
E138del	CC	≈2.5	yes (mouse)	- spontaneous CARD14 oligomerization - increased interaction with BCL10
E142G	CC	5	yes	
E142K	CC	4.03	yes	- increased interaction with MALT1 and BCL10, increased ERK/p38 activation
L150R	CC	1.79	no	
R151Q	CC	0.576	nd	
R151W	CC	1.766	nd	
L156P	CC	≈1.2	nd	- spontaneous CARD14 oligomerization
Q157P	CC	nd	nd	
R166H	CC	nd	nd	
H171N	CC	0.68	no	- increased interaction with MALT1 and BCL10, increased ERK/p38 activation
D176H	CC	2.78	no	- spontaneous CARD14 oligomerization
R179H	CC	1.38	no	
V191L	CC	1.02	no	
E197K	CC	1.667	nd	
S200N	CC	0.67	no	
L209P	CC	0.575	nd	
A216T	CC	nd	nd	
L228R	CC	1.5	nd	
D285G	CC	1.14	no	
M338V	CC	0.914	nd	
T420A	linker	0.663	nd	
E422K	linker	nd	nd	
R430W	linker	nd	nd	
R547S	linker	1	no	
V585I	linker	nd	nd	
T591M	PDZ	nd	nd	
I593N	PDZ	1.3	no	
S602L	PDZ	1.1	nd	
R682W	SH3	0.95	no	
G714S	SH3	1.02	no	
S802R	none	nd	nd	
R820W	GUK	nd	nd	
D973E	none	nd	nd	

*Summary of functional studies performed on CARD14 variants depicting ability to activate NF-κB and interaction with binding partners, BCL10 and MALT1*.

*CC, coiled-coil; GuK, guanylate-kinase domain; PDZ, PSD-95, discs-large, zona occludens-1 domain; SH3, Src homology 3 domain; O/E, overexpression*.

Seventeen CARD14 variants were tested for mRNA induction upon overexpression in the keratinocyte cell-line, HEK001 cells, by microarray or quantitative RT-PCR (9, 10) (Table 3). Four of these (p.E138A, p.G117S, p.E142K, p.E142G) displayed a pathogenic signature with strong induction of 13 genes including genes encoding CXCL8, IL-8, CSF2, SOD2, and TNFα, suggesting a role in skin inflammation. Additionally, CXCL8, CCL20, SOD2, and IL36γ transcripts were found increased in psoriatic skin from a GPP patient carrying the E138A mutation who also displayed an increased number of CARD14-positive keratinocytes by immunostaining. However, the other 13 variants (p.G714S, p.S200N, p.D176H, p.R179H, p.R38C, p.R62Q, p.I593N, p.H171N, p.R682W, p.L150R, p.V191L, p.D285G, p.R547S) failed to induce this pathogenic signature.

The CC domain of CARD11 is known to mediate its oligomerization upon activation (80). GoF mutations in the CC domain of CARD11 have been shown to trigger spontaneous protein aggregation and constitutive activation of NF-κB signaling leading to lymphoma (53). Berki et al. showed spontaneous CARD14 oligomerization for three variants located in the CC domain (p.E138A, p.D176H, and p.L156P), which did not occur in CARD14 WT or in the p.G117S variant, where the mutation is located outside the CC domain (38). Variants p.E138A, p.G117S, pE142K, and p.H171N were further characterized and show enhanced interaction with BCL10 and MALT1, thus resulting in increased NF-κB, ERK, and p38 MAPK activation (2, 71). This hyperactivity is reduced upon MALT1 protease inhibition with mepazine, thus suggesting that MALT1 treatment could benefit patients with CARD14-associated psoriasis.

Harden et al., showed CARD14 overexpression in dermal endothelial cells in psoriatic skin from patients harboring p.E138A and p.G117S mutations (5). This also correlated with enhanced NF-κB activity and proinflammatory gene induction, suggesting that CARD14 GoF in endothelial cells might also contribute to psoriasis pathogenesis. Intriguingly, the expression of CARD14 in aortic endothelial cells was also determined and warrants further investigation into the association of CARD14 mutation and associated cardiovascular defects in some psoriasis patients.

Recently, Danis et al showed that isolated primary keratinocytes from a patient with PRP type V, harboring three heterozygous variants (p.R547S, pR682W, p. R820W) and carrying a homozygous splice site variant (c.676-6G/A), displayed increased NF-κB activity compared to keratinocytes from healthy control (81). In this case, the analysis *ex vivo* of patient's cells carrying four variants do not allow to conclude about pathogenicity for each variant. However, it is interesting to note that at least two of these variants (p.R547S and pR682W) failed to induce increased NF-κB activity *in vitro* independently suggesting that *in vitro* assays might not be adequate to predict pathogenicity or that there is a cumulative effect occurring *in vivo*.

To conclude, there is strong evidence to consider p.E138A, p.E138del, p.G117S, p.E142K, p.E142G, and p.D176H as GoF variants based on their ability to trigger NF-κB activity and an inflammatory gene signature. p.E138A, p.E138del, p.D176H, and p.L156P could also be considered GoF due to their ability to trigger spontaneous CARD14 oligomerization and p.H171N based on its increased interaction with BCL10 (Table 1). It is however surprising that p.L156P and p.H171N trigger an increased CBM complex formation without increased NF-κB activation (38). This might be due to a sensitivity limitation of the assay used. Finally, p.E138A, p.E138K, p.E138del, p.G117S, p.E142K, p.E142G, p.L156P, and p.H171N could be considered as pathogenic since they are not found in healthy individuals according to public databases. Regarding other reported variants, more systematic functional studies and *ex vivo* studies from patient material are required to better understand how they could contribute to pathogenesis but they might be considered as variants associated with increased susceptibility to psoriasis rather than causal mutations.

## Physiological role of CARD14 in mouse psoriasis models

Findings in mouse models have further illuminated the physiological role of CARD14 in driving psoriatic skin disease. Heterozygous mice harboring a GoF mutation in the *Card14* gene at the glutamic acid residue E138 (*Card14*Δ*E138*) spontaneously develop a chronic psoriatic skin disease at 5 days-old (72). Deletion of this glutamic acid is synonymous to a human variant described in familial PRP family (12) and mutation of this residue has also been associated with the severe case of *de novo* GPP reported by Jordan and two sporadic cases of PRP with neonatal onset (58, 59). Like human disease the phenotype runs a chronic course in *Card14*Δ*E138*^+/−^ mice yet is mostly restricted to ear and tail tissue. Wang et al. subsequently generated *Card14E138A*^+/−^ and *Card14*Δ*Q136*^+/−^ mouse models and reported similar phenotypes to the *Card14*Δ*E138*^+/−^ mouse (82). Interestingly, the *Card14E138A*^+/−^ mouse had impaired survival rates compared to *Card14*Δ*Q136*^+/−^ mice, probably due to a more severe phenotype, in line with the enhanced potency of the human p.E138A mutation in *in vitro* studies. Though serendipitous incorrect editing by the CRISPR/Cas9 system generated the Card14ΔQ136 mutation, a similar human variant, p.Q136L, has been previously found in PRP type V (56). Histological analysis of *Card14* GoF mutant mice revealed typical hallmark features of human psoriatic skin disease with keratinocyte hyperproliferation, epidermal thickening and areas of parakeratosis and orthokeratosis, and with enlarged and increased number of blood vessels in the dermal compartment (72, 82). Other interesting features described in *Card14*Δ*E138*^+/−^ mutant mice include mild spongiosis within the epidermis, keratotic follicular plugging, which is a key feature of human PRP and the presence of neutrophil-rich microabscesses in the epidermis. Immune cell infiltration in *Card14* GoF mice consists of innate and adaptive immune arms, with significant high numbers of neutrophils, myeloid cells, as well as γδ- and αβ-T cells (72, 82).

Transcriptomic analysis of psoriatic tissue from *Card14*Δ*E138*^+/−^ ear pinnae revealed over 500 differentially expressed genes compared to wild-type littermates. These included genes encoding proinflammatory molecules of the adaptive (IL-17F, IL-20, IL-22, IL-23) and innate (IL-1 and IL-36 family cytokines, IL-19, IL-17C,) immune compartments. Neutrophil and Th17-chemoattractants, CXCL2 and CCL20 were also highly expressed as well as β-defensins and S100 proteins, which mirrors human psoriatic tissue. Indeed, antimicrobial peptides have a key role in driving human psoriatic skin disease and an increased copy number of β-defensins was previously associated with susceptibility to psoriasis (83). IL-17-responsive genes including small proline-rich proteins (SPRRs) and lipocalin-2 were also highly upregulated. Interestingly, and demonstrating the autoinflammatory and autoimmunity networks evident in this mouse model, genes encoding NOD2, Caspase-1, Caspase-4 and NLRP3 are also upregulated. This echoes the bimodal immune activation of psoriasis pathogenesis proposed by Christophers, showing innate neutrophils and macrophages orchestrating pathology alongside infiltrating Th17 cells, which maintain the chronic form of the disease (16). Interestingly, IL-38, an endogenous antagonist of IL-36 signaling was downregulated in psoriatic tissue from *Card14*Δ*E138* heterozygous mice, further suggesting a role for IL-36 signaling in this model. Enrichment analysis of the *Card14*Δ*E138*^+/−^ transcriptome compared to two published human datasets revealed high correlation of the murine phenotype with human plaque psoriasis.

Wang and colleagues demonstrated that the phenotype of *Card14*Δ*Q136*^+/−^ mice was ameliorated when crossed with *Il17a*- or *Rag1*-deficient animals, demonstrating an important contribution of T cells, in particular IL-17A-positive αβ T cells to the phenotype (82). Interestingly, *Card14*-deficient keratinocytes showed impaired responses to IL-17A and it was demonstrated that CARD14 interacts with the IL-17 receptor adaptor molecule, ACT1, suggesting that CARD14 is activated downstream of IL-17A in keratinocytes.

While CARD14 GoF is enough to drive full-blown psoriatic phenotype in mice, Tanaka and colleagues reported a requirement of CARD14 for psoriasiform disease development in other mouse models (84). The imiquimod mouse model is the most widely-used psoriasiform model for studying psoriatic skin disease due to the capability of TLR7 agonist imiquimod to activate plasmacytoid dendritic cells (pDCs), which are also implicated in mediating early events in human psoriasis pathogenesis (85, 86), *Card14*^−/−^ mice did not display thickening of the epidermis or immune cell infiltration of IL-17- and IL-22-secreting γδ T cells, characteristic of the imiquimod model. Therefore, CARD14-deficient mice were protected against developing psoriasiform disease in response to imiquimod compared to wild-type controls (84). These findings were corroborated by Wang and colleagues (82). Additionally, acanthosis caused by intradermal administration of IL-23 in wild-type mice mouse ears was diminished in CARD14-deficient animals and migration of IL-17- and IL-22-producing γδ T cells were significantly impaired in response to IL-23 in mice lacking functional CARD14 (84). This could be due to breaking of the chronic amplification loop in this model due to diminished CARD14-signaling within keratinocytes. However, the authors established bone-marrow chimeras and reported that *Card14*^−/−^ mice receiving a bone-marrow transplant from wild-type mice showed partial response to imiquimod, while conversely, irradiated WT recipient mice receiving bone-marrow from *Card14*^−/−^ donors showed decreased ear swelling in response to imiquimod compared to wild-type: wild-type chimeras. Tanaka's results suggest that CARD14 expression on radio-sensitive hematopoietic cells also contributes to psoriasiform disease in mice. Conversely, in the *Card14* GoF model, it was shown, using bone marrow chimeras, that transferring hematopoietic cells from *Card14*Δ*Q136*^+/−^ mice could not induce a psoriasiform phenotype in recipient WT mice, suggesting non-hematopoietic cells expressing mutant CARD14 are the drivers of disease pathogenesis in this model (82).Taken together these studies demonstrate a pivotal role for CARD14 in murine psoriasiform disease. Interestingly, while Tanaka and colleagues report that CARD14 is required for IL-23-induced disease and Wang demonstrated a role for CARD14 downstream of IL-17A in keratinocytes, the *Card14* GoF models show that CARD14 GoF mutation drives the pathogenic IL-23/IL-17 axis, suggesting that CARD14 is central to maintaining the chronic inflammatory cycle in murine psoriasiform disease. Furthermore, neutralization of IL-23p19 in Card14Δ*E138*^+/−^ mice significantly alleviated psoriatic skin, which mirrors the impact of targeting this cytokine subunit in clinical trials for human plaque psoriasis (87–89). These findings from murine psoriasis models would suggest that the CARD14 pathway is an important mediator of pro-inflammatory effects in human psoriatic skin disease also in patients lacking CARD14 GoF mutations.

## Discussion

Since the initial identification of GoF mutations of CARD14 as being responsible for psoriasis in two large kindreds and in a sporadic case of severe GPP, 44 missense, 4 splice site variants and 1 in frame deletion have been described in CARD14 in patients with several psoriatic skin disorders. One of the main complications is to understand how these variants contribute to disease progression. Among 49 variants reported as associated with psoriatic skin disease, 21 of them have never been tested functionally and eight others where only tested for NF-κB induction in overexpression systems. Interestingly, among the 28 variants tested for the ability to activate NF-κB, only six of these triggered increased NF-κB reporter activity more than two fold compared to overexpressed WT CARD14 (21%) (p.E138A, p.E138del, p.G117S, p.E142K, p.E142G, and p.D176H) (Table 3). Two other variants (p.L156P and p.H171N) did not show increased NF-κB induction but p.L156P displayed spontaneous CARD14 oligomerization (38). It remains to be clarified how this variant can contribute to psoriasis pathogenesis. Regarding the CARD14 variants that failed to induce enhanced NF-κB activity compared to WT CARD14, it is difficult to consider them as GoF and it remains to be elucidated by which mechanism they could be pathogenic. In this review, we have classified CARD14 reported variants as causal (Table 1) and associated (Table 2) based on the functional studies available but a better understanding of their mechanism of pathogenicity would help to classify pathogenic and non-pathogenic variants to comprehend the genetic contribution of CARD14 in psoriatic disorders. To date, the only common feature found between all pathogenic variants (with the exception of p.G117S) is their localization within the CARD14 coiled-coil domain, and they likely affect the autoinhibitory state of the protein. This might suggest that patients with variants in other regions of the CARD14 protein should undergo further genetic screening for potential cofactors.

Existence of cofactors have been reported in some studies, therefore it is possible that some CARD14 mutations only confer an increased risk of developing disease and other mutations are needed to develop psoriasis. Interestingly, in addition to HLA-Cw^*^0602, Spoerri and colleagues reported a kindred in which family members presented with PRP or psoriasis (42). While all family members had CARD14 mutations, the PRP sufferers had an additional frame-shift mutation in *DTX1*, a regulator of regulatory T cells, while the psoriasis-affected individual harbored a mutation in *NLRC5*, a molecule that activates NF-κB but also regulates MHC-I transcription (42). This kindred highlights how genetic cofactors may dictate how CARD14 mutations might contribute to diverse psoriatic entities.

Recently, another type of pustular skin type was also shown to be associated with CARD14. Acute exanthematous generalized pustular eruption (AGEP) is a rare generalized pustular skin rash that is triggered by an adverse reaction to drug administration (usually antibiotics but occasionally also anti-fungal or anti-malarial drugs), though sometimes in response to bacterial or viral infection (90, 91). The underlying disease mechanism of AGEP currently remains unknown but recent identification of *IL36RN* mutations being a causative factor in patients suggests a genetic and mechanistic connection between pustular psoriasis and AGEP (26, 92). Podlipnik et al. describe a 47-year old male patient presenting with AGEP and polyarthritis induced by dipyrone, a known AGEP culprit drug. It was discovered that the patient carried a heterozygous mutation (p.R430W) in the linker region of CARD14 (90). The authors predict that the variant is pathogenic based on bioinformatic analysis and its low frequency in healthy individuals from public database information (90).

The CARMA family have emerged as major mediators of both adaptive and innate immune responses and CARD14 was recently shown to have an important role in innate immune defense in keratinocytes in response to stimulation of the fungal Pattern Recognition Receptor, Dectin-1 and in response to bacterial ligands (73, 93). These *in vitro* data suggest that CARD14 plays a crucial role in modulating host defense at the skin barrier. However, this requires further clarification beyond *in vitro* studies. Utilizing CARD14-deficient mice in skin infection or barrier disruption models will lead to a greater understanding of how this molecule is activated and mounts an innate immune response to infection.

Interestingly, another CARMA molecule, CARD10, has recently turned out to be important in keratinocyte-induced immune responses. CARD10 was shown to be highly expressed in proliferating keratinocytes, whereas CARD14 was shown to be expressed at low levels in proliferating keratinocytes but induced upon their differentiation (6). This balance between CARD10 and CARD14 might be important in the context of psoriasis where keratinocyte differentiation processes are known to be dysregulated. Additionally, CARD10 was shown to regulate NF-κB in endothelial cells, for example in response to angiotensin (94) and potentially GoF mutation in CARD14 might destabilize a potential CARD10/CARD14 balance, and thus could contribute to cardiovascular comorbidities.

Beyond keratinocytes and endothelial cells, expression of CARD14 was also described in bone marrow-derived hematopoietic cells in mice. It will be of interest to determine the contribution of these cell types, harboring CARD14 mutations, to human psoriasis pathogenesis. To date, CARD14 GoF mutation studies have been limited to overexpression systems, primarily in cell-lines. It can be anticipated that CRISPR/Cas9 technology will aid in further elucidating the function of CARD14 GoF mutations in different cell types from *in vivo* models and in keratinocyte cell-lines. Little is known, also about the function of CARD14 in other tissue types.

CARD14 has previously been shown as a strong inducer of IL-36γ in primary keratinocytes highlighting a link between *CARD14* GoF mutations and the IL-36 cytokines responsible for GPP pathogenesis. Interestingly, plaque and pustular forms of psoriasis can present concurrently (16, 95) and Christophers and colleagues propose a bimodal model of immune activation within psoriasis with alternate activation of autoinflammatory and autoimmune networks (16). Due to the shared characteristics of psoriasis entities caused by *CARD14* and *IL-36RN* mutation Akiyama and colleagues propose that these disease subtypes should be grouped together as autoinflammatory keratinization diseases (AIKDs) (96, 97). The main defining factors of AIKDs is that the primary inflammatory sites occur at the epidermis and upper dermis resulting in hyperkeratosis with mixed autoinflammatory and autoimmunity circuits driving pathogenesis. Therefore, *IL-36RN*-associated GPP, and CARD14-mediated pustular psoriasis, PRP type V and familial keratosis lichenoides chronica (KLC) caused by *NLRP1* mutation, can be considered AIKDs.

In *Card14*Δ*E138*^+/−^ mice both autoinflammatory and autoimmune networks were upregulated at the transcript level suggesting that CARD14 GoF mutation drives both adaptive and innate immune networks, which might explain why mutant variants of CARD14 have been associated with both plaque and pustular forms of psoriasis. Disruption of the adaptive immune response by TNF and IL-12/IL-23 blockade has proven successful in case reports of patients harboring CARD14 GoF mutations and findings from mouse models suggest that targeting the IL-23/IL-17 axis would also be beneficial for patients. Utilising an NF-κB inhibitor was also favorable in Card14 GoF mice (82) and specific inhibition of MALT1 or CARD14, itself, might also be attractive therapeutic options in the future.

## Author contributions

All authors listed have made a substantial, direct and intellectual contribution to the work, and approved it for publication.

### Conflict of interest statement

The authors declare that the research was conducted in the absence of any commercial or financial relationships that could be construed as a potential conflict of interest.
